# Supplementary material for: Chromatin accessibility changes at intergenic regions are associated with ovarian cancer drug resistance
Source: Clin Epigenetics. 2021 Jun 5;13:122. doi: 10.1186/s13148-021-01105-6 (PMC8180030; doi:10.1186/s13148-021-01105-6)
Supplement: Supplementary file 1 — Additional file 1. Supplementary Figures and Tables. [file 13148_2021_1105_MOESM1_ESM.pdf]

Supplementary Figure 1

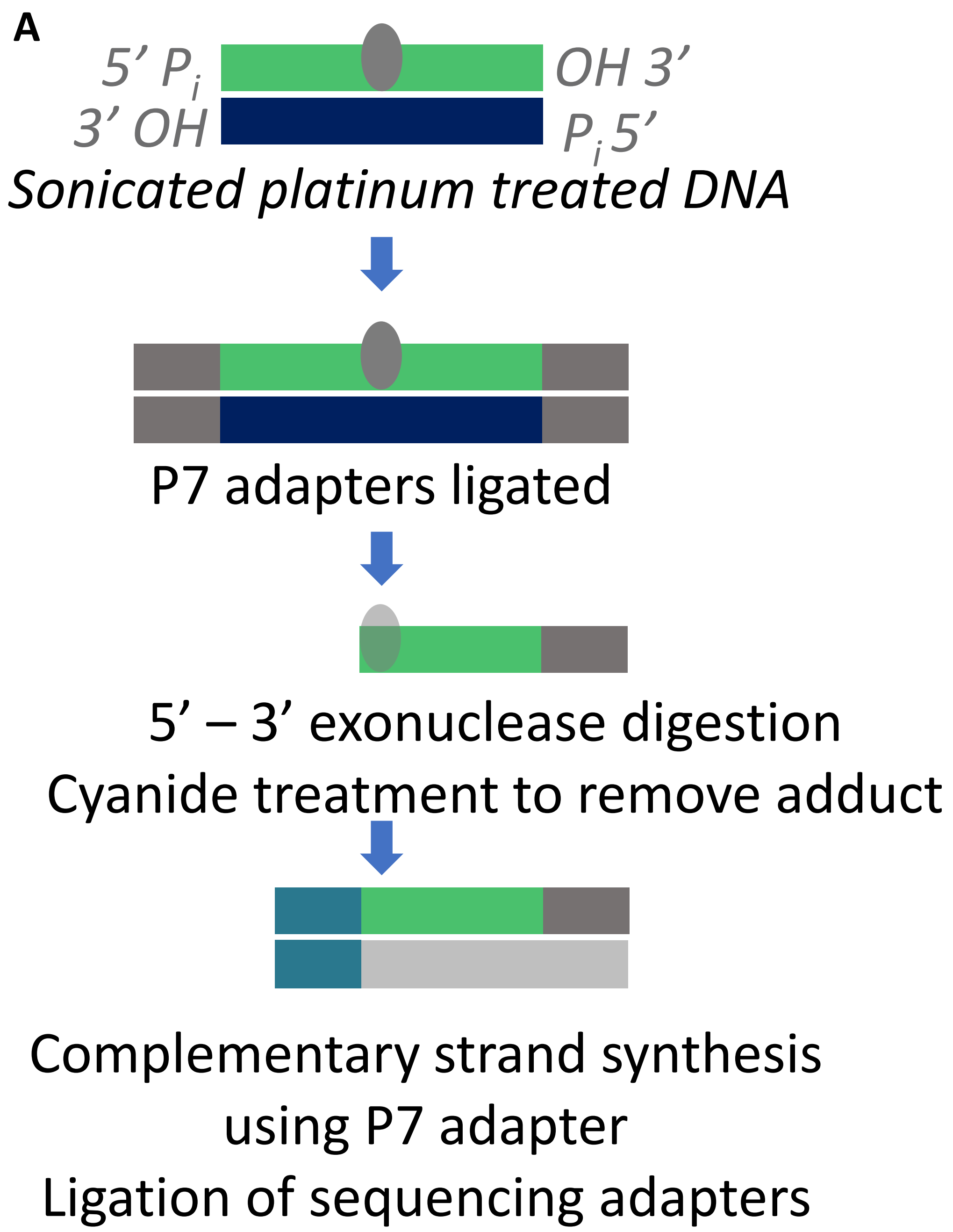

DNA isolated from cisplatin treated cells is sonicated and following P7 adapter ligation is digested with 5′ to 3′ lambda exonuclease with RecJF and SSB. The presence of a cisplatin adduct stops the exonuclease digestion. Following removal of the platinum adduct by cyanide treatment, complementary strands are synthesizes using the P7 adapters and next generation sequencing libraries prepared.

Supplementary Figure 2. Differential Gene expression across cell line pairs.

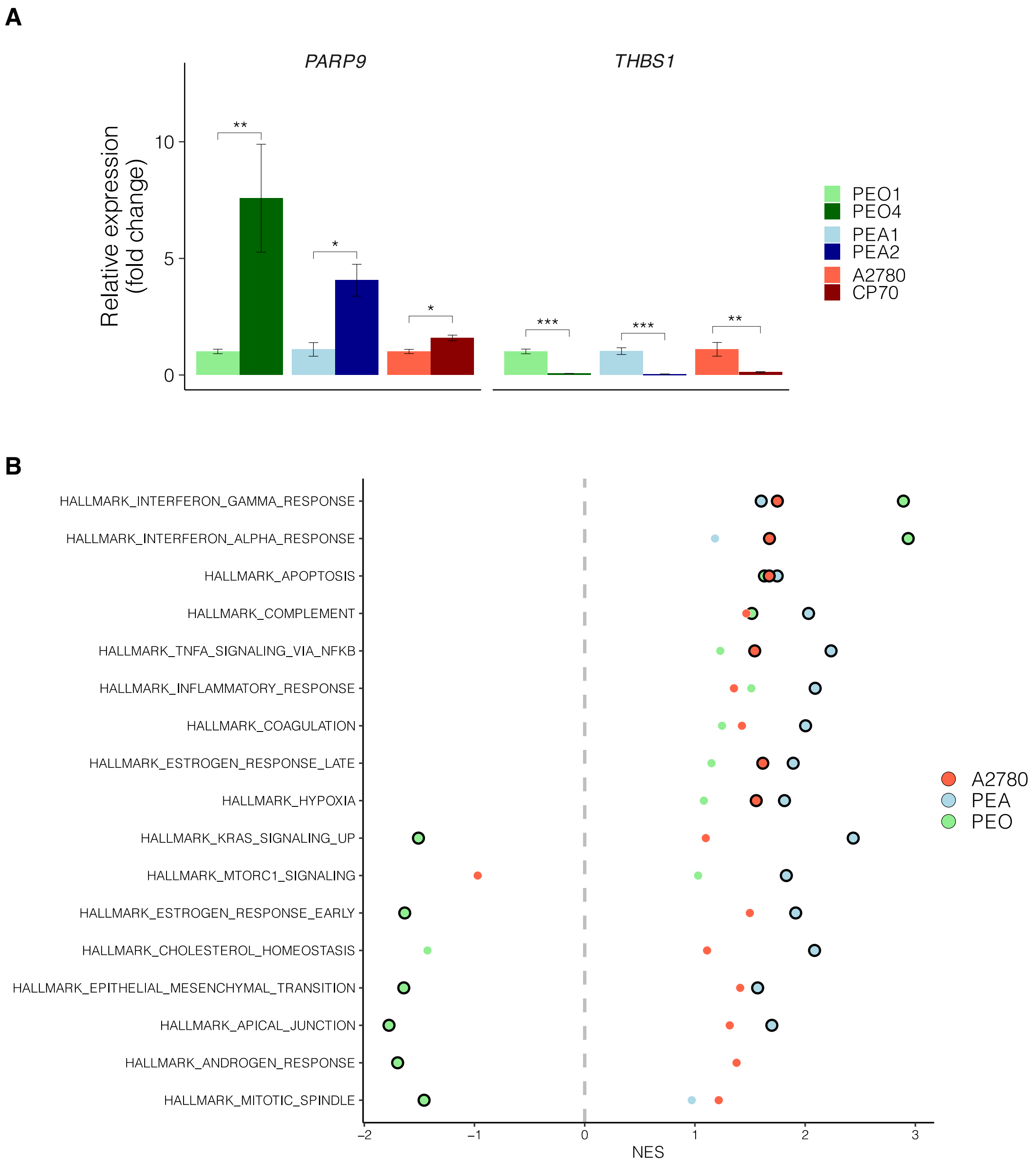

A) Expression of *PARP9* and *THBS1* in paired, cisplatin sensitive and resistant ovarian cancer cell lines. Relative expression was calculated by RT-QPCR from 3 biological replicates for each line. Fold change was calculated using the ΔΔCt method, using *ACTB* and *GAPDH* as housekeeping genes. Error bars show SEM. * = $P < 0.05$, ** = $P < 0.01$, *** = $P < 0.001$. **B**) Gene set enrichment analysis using hallmark gene sets using *t* statistics from limma for each sensitive-resistant comparison. X axis shows normalised enrichment score. Outlines indicate a significantly altered gene set (FDR < 0.05).

Supplementary Figure 3

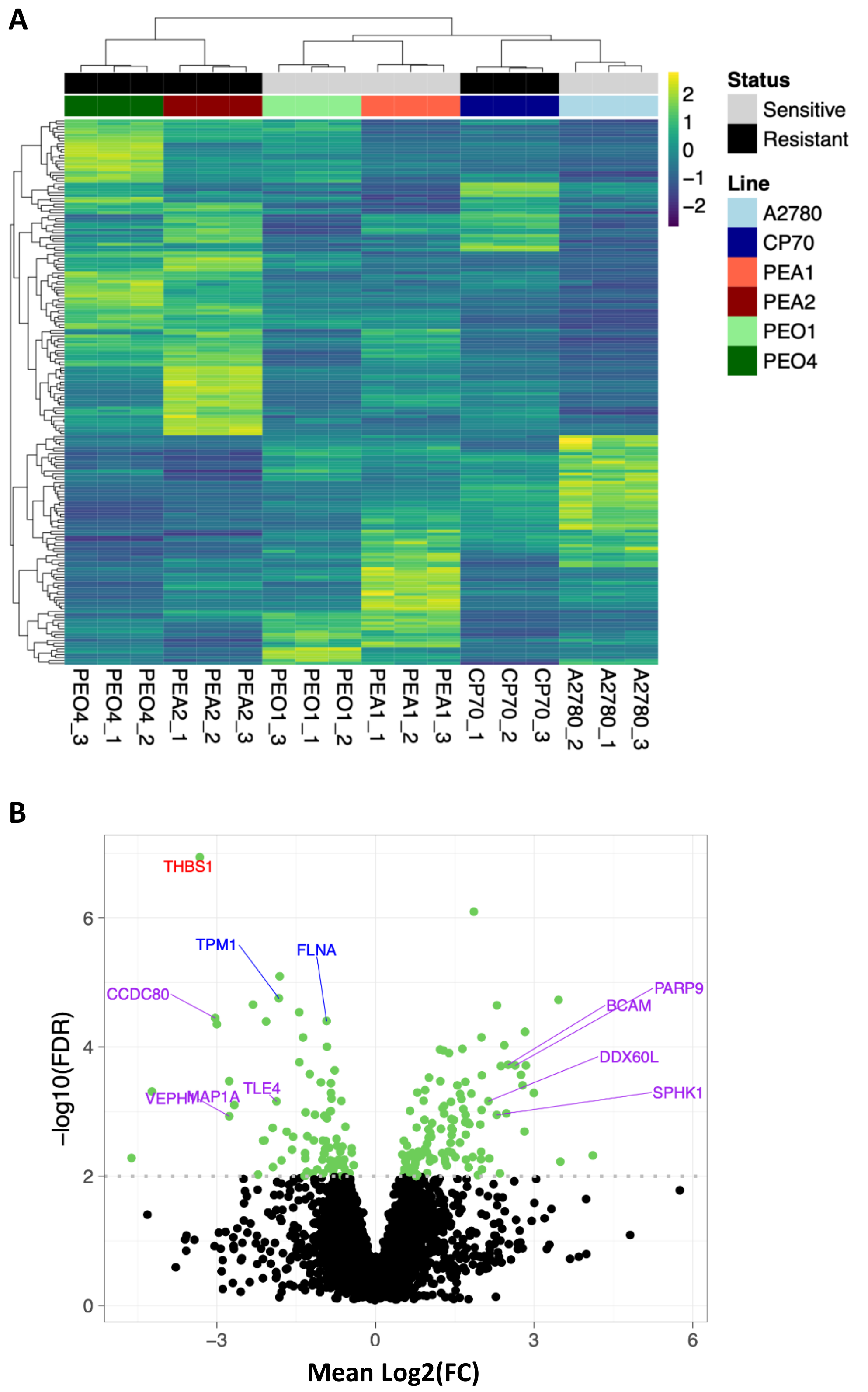

Volcano plots and heatmap of 617 DE genes as defined by rank product analysis. **A**) Heatmap of gene expression in replicate, for DE genes. Dendrogram shows relationship between samples as defined by unsupervised hierarchical clustering. **B**) Volcano plot of gene expression between sensitive and resistant lines. DE genes (FDR <0.01) shown in green. Log2FC is mean change within each pair. Genes showing independent differential expression in all three lines labelled in purple. Genes defined as hypermethylated and downregulated in Zeller et al. 2011 labelled in blue. Genes in both the Zeller et al. 2011 set and the intersection labelled in red. Dashed lines show significance thresholds.

Supplementary Figure 4: Example browser shots of ATACseq data at upregulated and down regulated genes

THBS1 - Downregulated

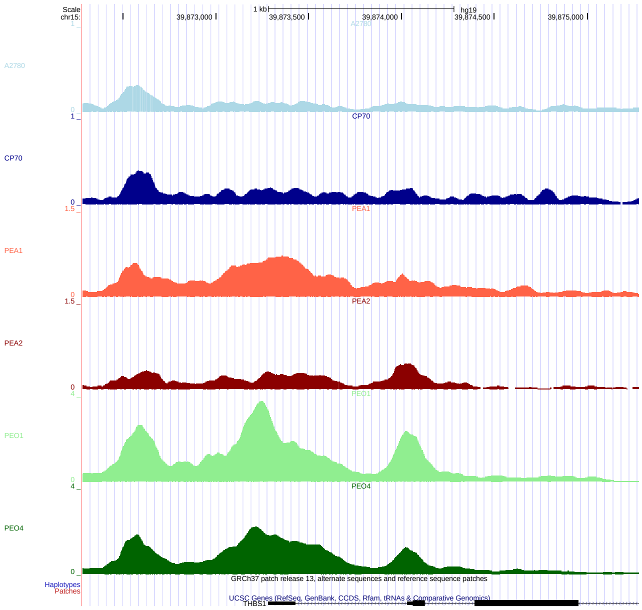

PARP9 - Upregulated

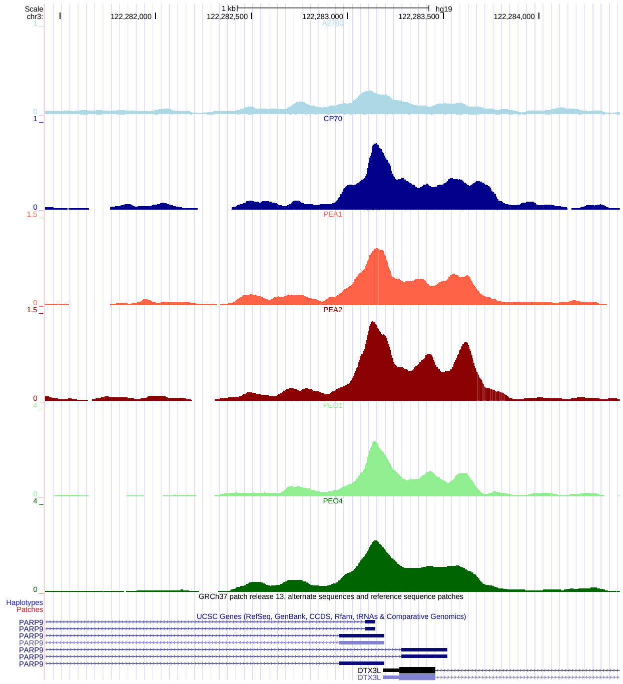

Supplementary Figure 5. Enrichment for chromatin states in COREs lost and gained in PEO4 compared to PEO1.

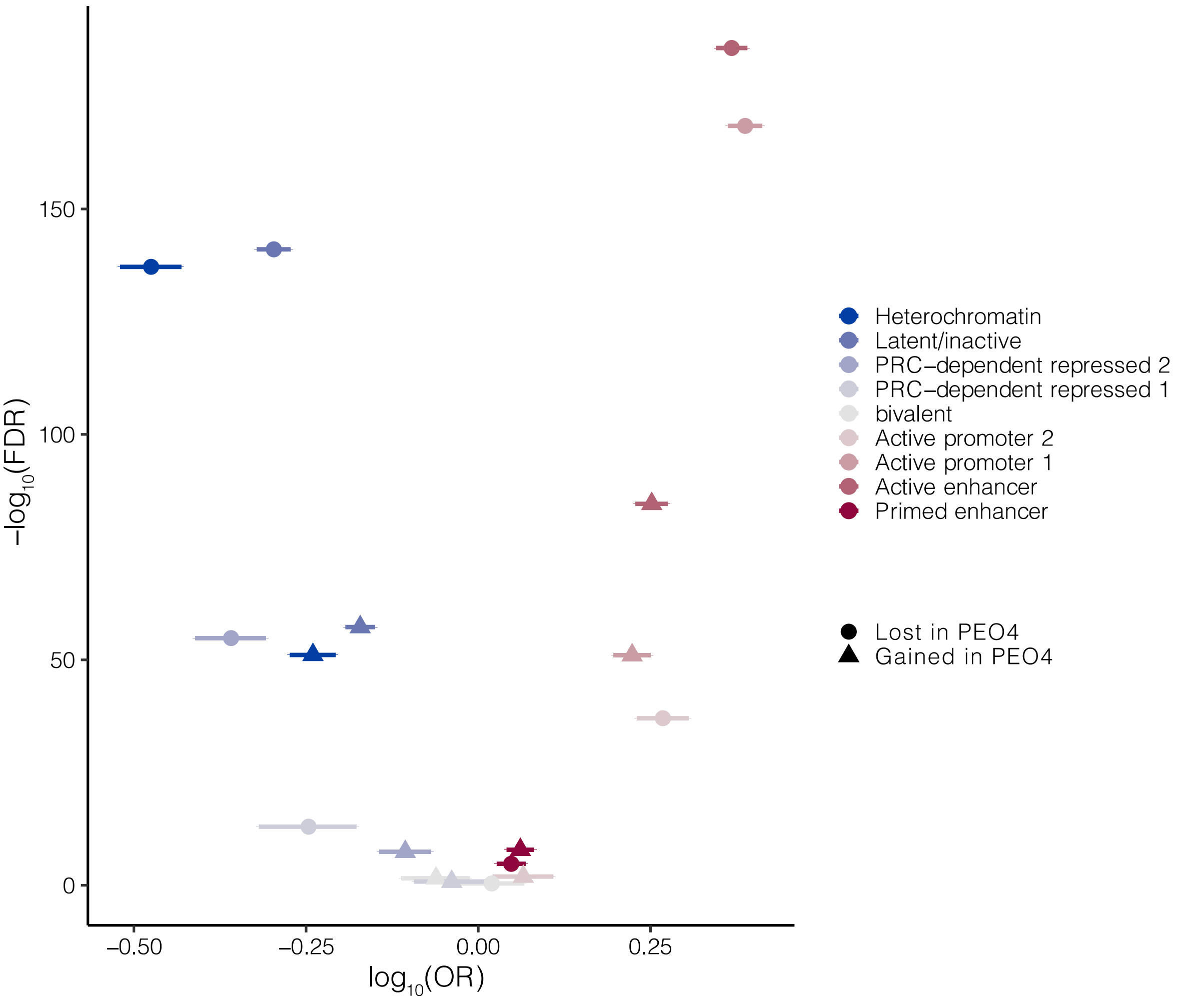

X axis shows $\log_{10}$ odds ratio from Fisher's exact test comparing proportion of regions associated with a given chromatin state, in COREs lost/gained in PEO4 compared to PEO1, against the proportion of regions falling outside COREs which are associated with that state. Error bars show 95% confidence interval.

Supplementary Figure 6. Examples of COREs identified with differential accessibility A. Sox9, PEO 4 versus PEO 1, B. FN1, PEA1 versus PEA2, C. AMIGO2, A2780 versus A2780/cp70

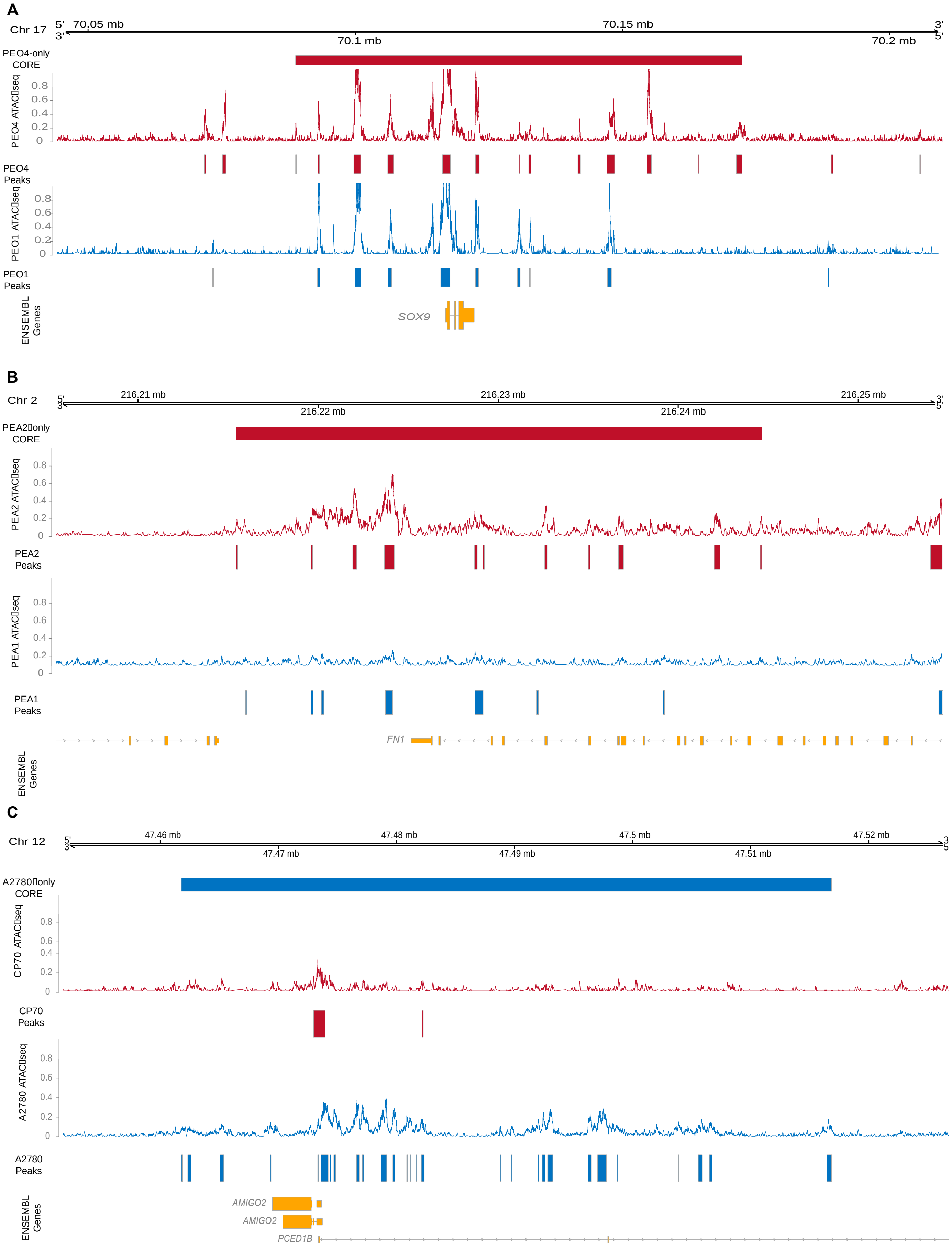

Supplementary Figure 7

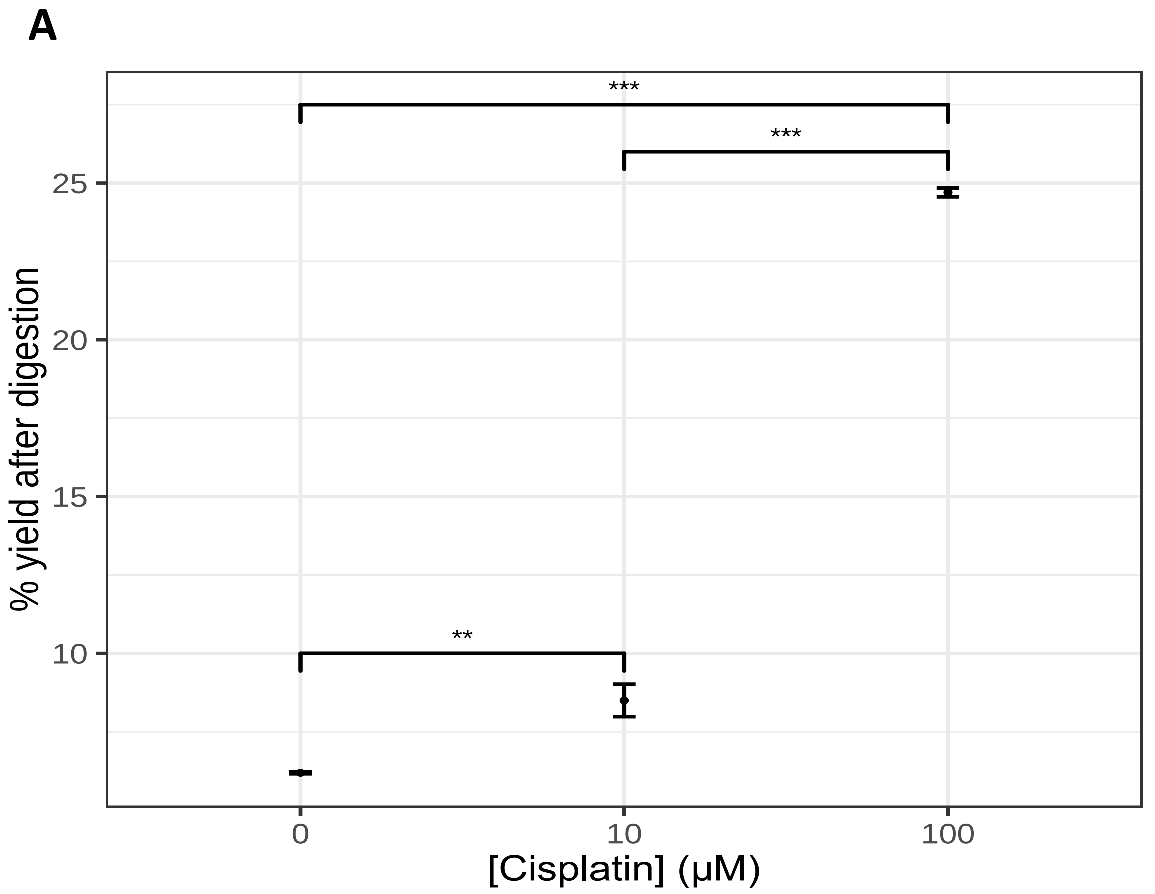

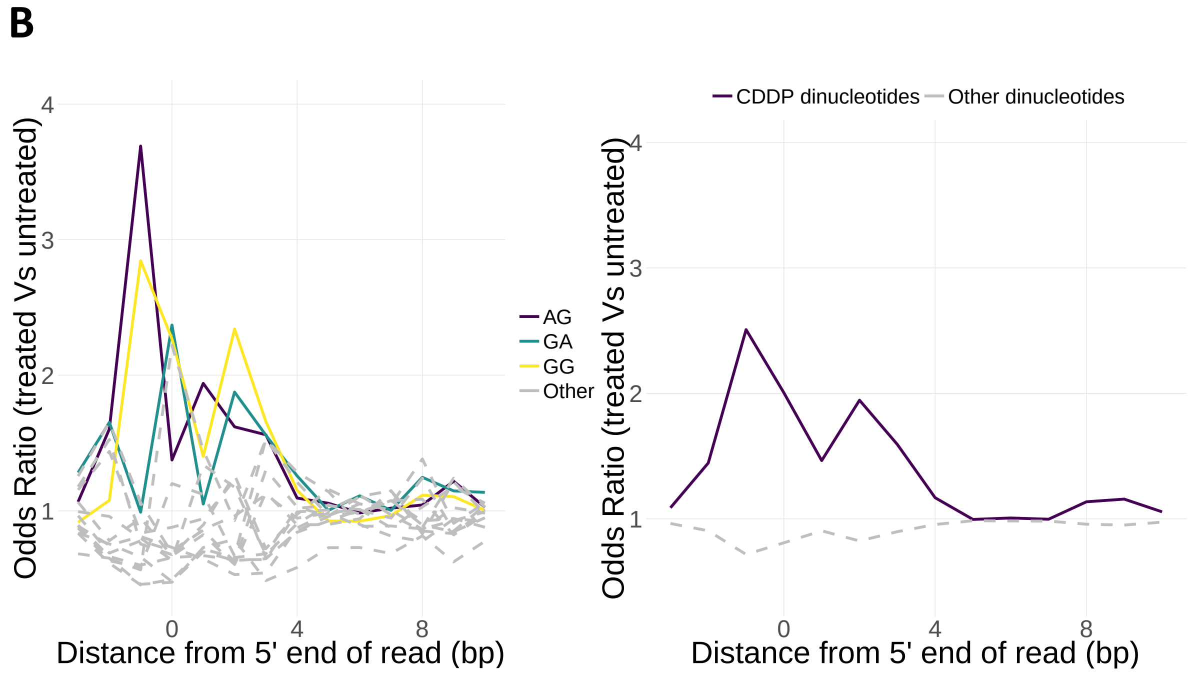

A. Percent yield of DNA following Exonuclease digestion of DNA isolated from A2780 cells treated with 0, 10μM and 100μM cisplatin for 5 hours (p<0.01, ***p<0.001). B Enrichment for platinum target purine dinucleotides at the 5' end of Pt-exo-seq reads after treatment of A2780 cells with 100μM cisplatin as Odds Ratio from Fisher Exact test.**

Supplementary Figure 8

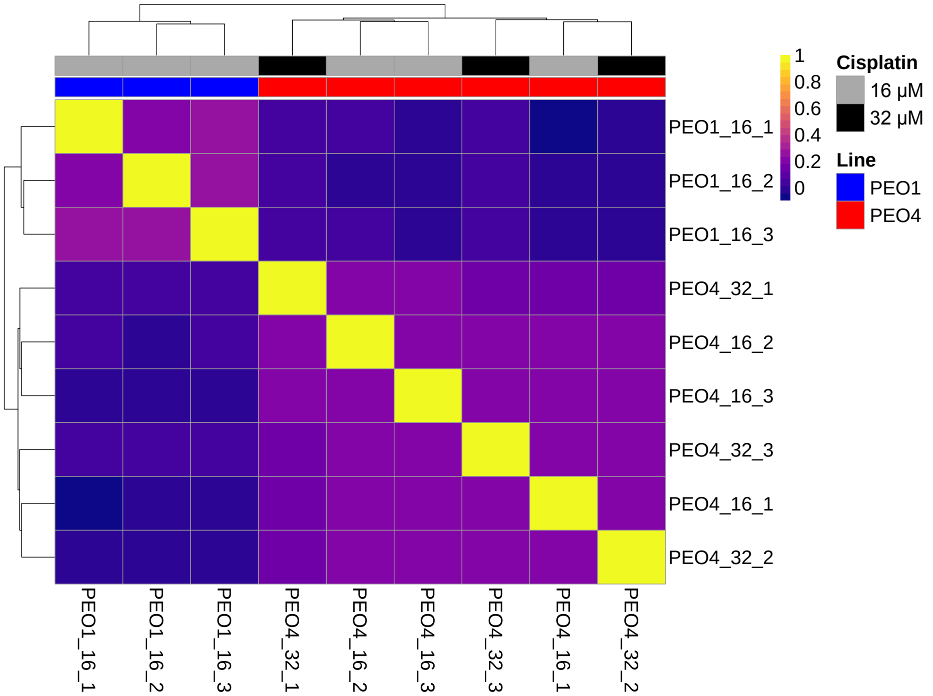

Correlation of Pt-exo-seq signal between each replicate. Heatmap shows Spearman's correlation coefficient. Dendrogram reflects unsupervised hierarchical clustering of replicates. Spearman's correlation coefficients calculated for all 61,590 1 Kb windows passing thresholds for analysis in ATAC-seq. Cells were treated for 5 hours with the indicated dose of cisplatin.

Supplementary Figure 9

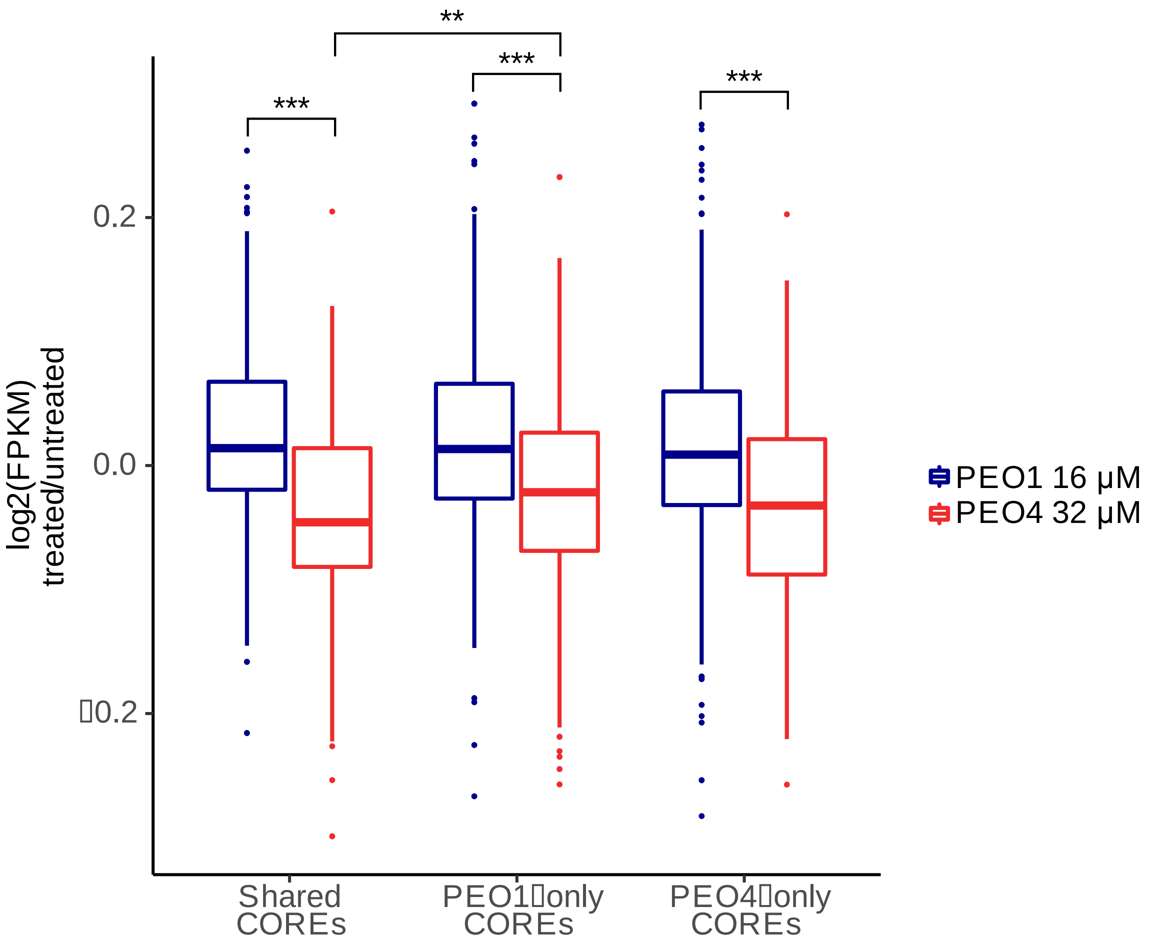

Cisplatin damage in COREs in the PEO pair. Cisplatin adduct formation, as measured by Pt-exo-seq, at COREs shared between PEO1 and PEO4 (246), COREs lost in PEO4 (480), and COREs gained in PEO4 (315). Cell lines were treated with doses shown to induce similar total genomic adducts as measured by ICP-MS (PEO1 16 μM, PEO4 32 μM). Centre lines show the mean, lower and upper hinges correspond to the first and third quartiles, and whiskers extend to 1.5(IQR). Points are shown for values outside this range. * *= $P < 0.01$, *** = $P < 0.001$, Wilcox test.

Supplementary Figure 10

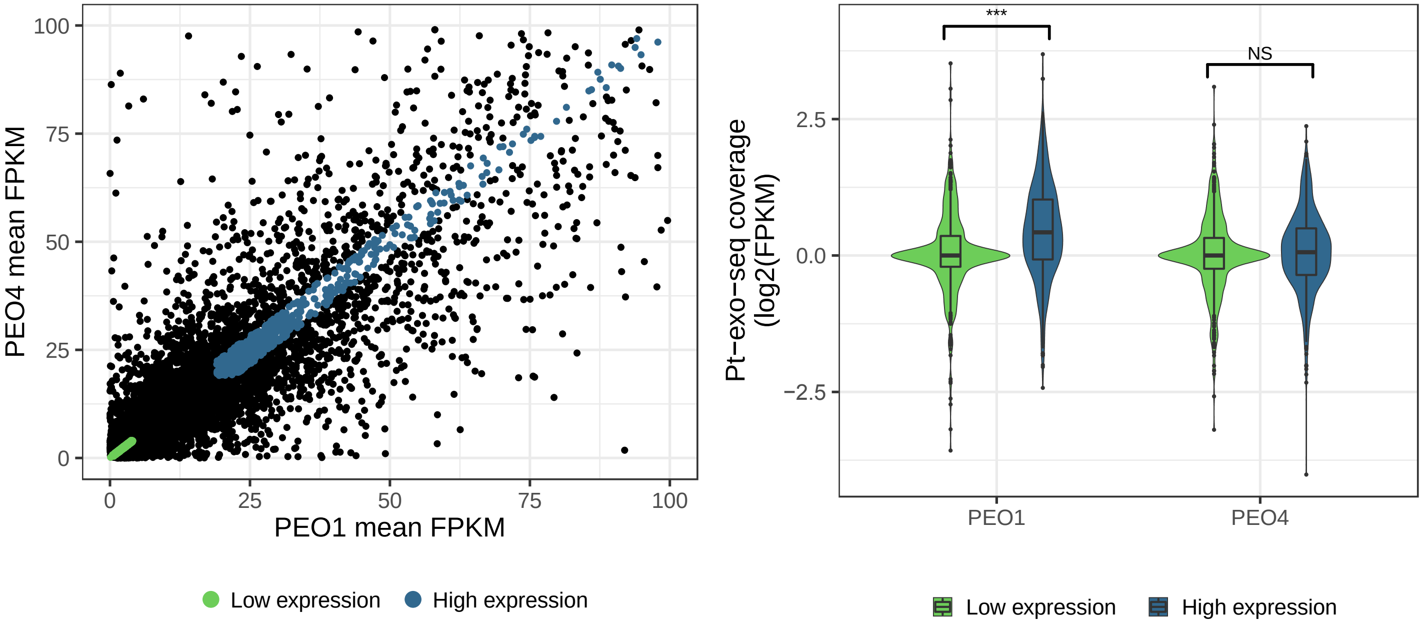

Relationship between Pt-exo-seq signal and gene expression in PEO1 and PEO4. **A**) Dot plot of gene expression in PEO1 and PEO4, showing genes selected for Pt-exo-seq coverage analysis based on equally high or low expression across lines. **B**) Pt-exo-seq signal in 2 Kb around TSS' of genes showing equally high or low expression in PEO1 and PEO4. 494 low expression genes and 557 high expression genes were used in this analysis. Genes were selected by being in the highest or lowest quartiles for both PEO1 and PEO4, with a difference in expression in lowest quartile. (*** = $p < 0.001$, t test)

Supplementary Table 1 Characteristics of cell line pairs used. Information on treatment and derivation is from the European Collection of Authenticated Cell Cultures. Fold differences in cisplatin sensitivity are from the mean of 3 reported IC_{50} values for the A2780 and PEO pairs.

Cell line	Derivation	Treatment	Collection	Average reported fold difference in platinum sensitivity	Histology	Pt DNA adduct repair	MLH1 status	BRCA1 status
A2780	Tumour tissue from untreated patient	NA	Before treatment	9.5	E, CC	++	+	WT
CP70	Derived from A2780 by exposure to increasing concentrations of cisplatin	*in vitro* cisplatin	Derived from A2780		E, CC	++	-	WT
PEO1	malignant effusion, peritoneal ascites, poorly differentiated serous adenocarcinoma	cisplatin, 5-fluorouracil and chlorambucil	Before resistance	7.7	HGS	-	NA	WT, LOH?
PEO4	malignant effusion, peritoneal ascites, poorly differentiated serous adenocarcinoma	cisplatin, 5-fluorouracil and chlorambucil	Post development of resistance		HGS	++	NA	WT, LOH
PEA1	malignant effusion from pleural cavity, poorly differentiated adenocarcinoma	NA	Prior to cisplatin and prednimustine	4.7	HGS	++	NA	WT, LOH
PEA2	malignant effusion from peritoneal ascites, poorly differentiated adenocarcinoma	Cisplatin and prednimustine	On relapse		HGS	++	NA	WT, LOH

Supplementary Table 2. The number of windows falling within each genomic class identified

Class	No.Windows
CpG	11500
promoter-TSS	38565
exon	37597
intron	783018
TTS	34658
3' UTR	25907
5' UTR	3822
intergenic	1094294
ERV	879
SINE	430028
LINE	701598
LTR	293848
Simple_repeat	28442
non-coding	13051

Supplementary Table 3. Summary of analysis of differential gene expression between each paired sensitive and resistant line. DE = (log2FC ± 2 and FDR <0.05).

Pair	Total genes analysed	Total Genes DE	Downregulated	Upregulated
A2780	10,290	998	390	608
PEO	10,956	962	510	452
PEA	10,807	2013	920	1093

Supplementary Table 4 Summary of numbers of COREs called by CREAM in each line. COREs unique to a line indicate number of COREs lost or gained in the resistant line, while shared in pair are those present in both pair.

Line	COREs	Unique COREs	Shared in pair
A2780	1030	847	190
CP70	358	192	
PEA1	744	601	148
PEA2	811	667	
PEO1	715	480	246
PEO4	537	315	

Supplementary Table 5 Enzymes and buffers used for Pt-exo-seq library preparation

Enzyme	Usage	Source	Code
Micrococcal Nuclease I	Digestion of chromatin for laddering	NEB	M0247S
Buffer 2	Enzymatic reactions	NEB	B7002S
dNTPs	PCR, end repair, complementary strand synthesis		
ATP	ATP dependent enzymatic reactions	NEB	P0756S
T4 DNA polymerase	Blunt end generation	NEB	M0203S
DNA Polymerase I (Klenow fragment)	Blunt end generation	NEB	M0210S
T4 Polynucleotide Kinase	Blunt end generation	NEB	M0201S
T4 DNA Ligase	Ligation of sequencing adapters	NEB	M0202S
phi29 DNA Polymerase	Nick repair following adapter ligation	NEB	M0269S
Lambda Exonuclease	5' - 3' exonuclease digestion	NEB	M02062S
Lambda Exonuclease Buffer	-	NEB	B0262S
RecJF Exonulease	5' - 3' exonuclease digestion	NEB	M0264S
Single-Stranded DNA Binding Protein	Aid digestion of single stranded DNA	Promega	M3011

Supplementary Table 6 Sequences of oligonucleotides used in Pt-exo-seq library preparation

Oligo name	5' modification	Barcode	Sequence
P7 adapter R	[Phos]	-	GTGACTGGAGTTCAGACGTGTGCTCTTCCGATC
P7 adapter F	-	-	GATCGGAAGAGCACACGTCT
P5 adapter R	-	-	AGATCGGAAGAGCG
P5 adapter F	-	-	TACACTCTTTCCCTACACGACGCTCTTCCGATCT
P7 primer	-	-	GACTGGAGTTCAGACGTGTGCT
Universal reverse primer	-	-	AATGATACGGCGACCACCGAGATCTACACTCTTTCCCTACACG*A
PCR Primer index 1	-	CGTGAT	CAAGCAGAAGACGGCATACGAGATCGTGATGTGACTGGAGTTCAGACGTGTGC*T
PCR Primer index 2	-	ACATCG	CAAGCAGAAGACGGCATACGAGATACATCGGTGACTGGAGTTCAGACGTGTGC*T
PCR Primer index 3	-	GCCTAA	CAAGCAGAAGACGGCATACGAGATGCCTAAGTGACTGGAGTTCAGACGTGTGC*T
PCR Primer index 4	-	TGGTCA	CAAGCAGAAGACGGCATACGAGATTGGTCAGTGACTGGAGTTCAGACGTGTGC*T
PCR Primer index 5	-	CACTGT	CAAGCAGAAGACGGCATACGAGATCACTGTGTGACTGGAGTTCAGACGTGTGC*T
PCR Primer index 6	-	ATTGGC	CAAGCAGAAGACGGCATACGAGATATTGGCGTGACTGGAGTTCAGACGTGTGC*T
PCR Primer index 7	-	GATCTG	CAAGCAGAAGACGGCATACGAGATGATCTGGTGACTGGAGTTCAGACGTGTGC*T
PCR Primer index 8	-	TCAAGT	CAAGCAGAAGACGGCATACGAGATTCAAGTGTGACTGGAGTTCAGACGTGTGC*T
PCR Primer index 9	-	CTGATC	CAAGCAGAAGACGGCATACGAGATCTGATCGTGACTGGAGTTCAGACGTGTGC*T
PCR Primer index 10	-	AAGCTA	CAAGCAGAAGACGGCATACGAGATAAGCTAGTGACTGGAGTTCAGACGTGTGC*T
PCR Primer index 11	-	GTAGCC	CAAGCAGAAGACGGCATACGAGATGTAGCCGTGACTGGAGTTCAGACGTGTGC*T
PCR Primer index 12	-	TACAAG	CAAGCAGAAGACGGCATACGAGATTACAAGGTGACTGGAGTTCAGACGTGTGC*T
PCR Primer index 13	-	TTGACT	CAAGCAGAAGACGGCATACGAGATTTGACTGTGACTGGAGTTCAGACGTGTGC*T
PCR Primer index 14	-	GGAACT	CAAGCAGAAGACGGCATACGAGATGGAACTGTGACTGGAGTTCAGACGTGTGC*T
PCR Primer index 15	-	TGACAT	CAAGCAGAAGACGGCATACGAGATTGACATGTGACTGGAGTTCAGACGTGTGC*T
Illumna Library quantification F	-	-	AATGATACGGCGACCACCGA
Illumna Library quantification R	-	-	CAAGCAGAAGACGGCATACGA*G
